# Primary healthcare utilisation and associated factors among post-treatment prostate cancer survivors in Tanzania: a cross-sectional study

**DOI:** 10.3332/ecancer.2026.2143

**Published:** 2026-06-09

**Authors:** Rabson R Nakey, Gideon P Kwesigabo, Monica C Shabani, Yemela K Ndibalema, Aurelia J Temba

**Affiliations:** 1 Department of Epidemiology and Biostatistics, MUHAS, United Nations Road, Ilala, PO Box 65000, Dar es Salaam, Tanzania; 2 Oncology Wing, Besta Super Specialized Polyclinic, Plot 122, Tunisia Road, Kinondoni, PO Box 5596, Dar es Salaam, Tanzania; 3 Mwananyamala Regional Referral Hospital, Mwananyamala, Mwinyijuma Road, Kinondoni, PO Box 61665, Dar es Salaam, Tanzania

**Keywords:** prostate cancer survivor, primary healthcare, primary healthcare utilisation, post-treatment

## Abstract

Prostate cancer survivorship is becoming an increasingly important issue in Tanzania, especially because follow-up care is still heavily centralised in a few specialised centres. This model is difficult to sustain in a country with a limited number of oncologists and specialised facilities. At the same time, there is growing recognition both globally and locally that primary healthcare (PHC) should play a major role in supporting cancer survivors. With this in mind, the present study set out to determine the proportion of prostate cancer survivors who actually use PHC services after completing active treatment, and to identify the factors that influence this pattern of care. A cross-sectional study was conducted at Besta Super Specialised Polyclinic in Dar es Salaam, recruiting 213 survivors who completed radiotherapy between January 2021 and December 2023. Data were collected via medical record reviews and telephone interviews using the European Organisation for Research and Treatment of Cancer Quality of Life Questionnaire Prostate Cancer Module to assess symptom burden.

The study found that 69.0% of survivors utilised PHC services, primarily at district hospitals (82.3%). Rural residence was the strongest predictor, with rural survivors over ten times more likely to access care than their urban counterparts. Higher education, early-stage diagnosis and severe treatment or sexual side effects also significantly increased utilisation. Interestingly, mild urinary symptoms were associated with higher PHC use compared to severe cases.

These results suggest that a significant proportion of prostate cancer survivors in Tanzania are already engaged with PHC as a source for addressing survivorship issues, particularly those in rural settings with high symptom burdens. Given the persistent gaps and challenges in accessing specialised cancer services, it becomes even more important to recognise, understand and strengthen the role of PHC facilities as an essential part of the country’s cancer care system.

## Introduction

Prostate cancer is the leading malignancy among men in Tanzania and reflects the increasing incidence trends reported globally across many low- and middle-income countries [1, 2]. With the expansion of diagnostic capabilities and treatment modalities, especially radiotherapy (RT), there is an increasing survival of men. While improved survival is a huge success, this raises a new challenge for the healthcare system, an increasing population of survivors who are in need of long-term management for treatment-related late effects, comorbidities and psychosocial support [3, 4].

The Tanzanian healthcare system is organised into a three-tier pyramidal structure primary, secondary and tertiary to manage the delivery of health services across the country [5, 6]. For cancer survivors, this structure often functions as a vertical referral hierarchy, where the specialised nature of oncology services necessitates that patients move upward from local primary facilities to centralised institutions [6, 7]. Patients are typically referred from rural areas to specialised urban ‘hubs’ for acute treatment, including private facilities such as Besta Hospital in Dar es Salaam or national centers like the Ocean Road Cancer Institute, Muhimbili National Hospital and zonal hospitals which house the country’s advanced RT and systemic therapy capabilities [6, 8].

Once acute treatment is completed, survivors are frequently not formally referred back to primary healthcare providers for their long-term survivorship care [9, 10]. This is largely due to a weak and often confusing referral system that lacks standardised protocols for downward transition, leaving a significant gap in the coordination between oncology specialists and local practitioners [7, 11]. Instead of being reintegrated into their local communities’ health networks, survivors are often maintained on scheduled, routine follow-up at the tertiary center or specialised hub [8, 10].

The centralisation creates a ‘disconnection’ in the care continuum, as local primary health facilities frequently lack the necessary patient information and specialised training to manage post-treatment needs or conduct routine screenings [5, 7, 12]. Without a formal clinical handover, survivors must independently navigate local dispensaries/health centers or district hospital for regular medical needs and treatment-related toxicities, often without the benefit of a clear survivorship care plan or integrated follow-up strategy [9, 10, 13]. This model places a heavy burden on survivors, particularly those in rural settings who must continue traveling long distances to urban centers for routine monitoring that could potentially be managed closer to home if the referral system were more bidirectional [5, 6].

Currently, international oncology guidelines are increasingly advocating for a shift away from acute, specialist-led models of care to shared care approaches that include the involvement of primary healthcare (PHC) providers. This is especially important in resource-constrained settings like Tanzania, where the lack of oncologists and specialised centers makes a specialist-only follow-up model unsustainable. Moreover, shifting care to the primary level is not only part of a strategy to decongest tertiary hospitals but is equally important in ensuring equitable access to continued care among survivors residing in rural and under-resourced areas [14].

However, the utilisation of PHC services is usually driven by the immediate health needs of the survivors. After active treatment, men more often continue to suffer from late effects such as urinary, bowel and sexual dysfunction [15]. These symptoms can be chronic and distressing, significantly influencing health-seeking behaviour. However, the direction of this influence remains unclear regarding whether a high symptom burden drives survivors to seek immediate relief at the nearest primary facility or if the severity of complications compels them to bypass local clinics in favour of tertiary specialists. Understanding how different domains of symptom burden shape these choices is vital for ensuring PHC facilities are clinically equipped to manage the specific complications survivors present with.

Despite these considerations, post-treatment care in Tanzania remains centralised. There is a dearth of empirical evidence to show whether survivors currently use PHC services to address their follow-up needs or bypass these health facilities altogether [16]. Most literature comes from high-income countries with established referral systems; thus, generalising findings to Tanzania, where there are different sociodemographic characteristics, would also not be appropriate, such as rural residence and lower levels of education, which play a critical role [17, 18]. Therefore, this study sought to establish the extent of post-treatment PHC use and its sociodemographic and clinical correlates among prostate cancer survivors in Tanzania.

## Methodology

### Study design and setting

A cross-sectional study was conducted at Besta Super Specialised Polyclinic, located at Plot 122, Tunisia Road, Kinondoni district, Dar es Salaam, Tanzania. The clinic is a private, multi-disciplinary facility that serves as a referral center for oncology, treating an average of 540 cancer patients annually, with prostate cancer accounting for approximately 55% of cases. The facility provides 3D RT and chemotherapy, bridging the gap between acute cancer treatment and long-term survivorship care in the country.

Besta Super Specialised Polyclinic serves a socioeconomically diverse patient population as one of the major accredited provider for the National Health Insurance Fund (NHIF). By accepting both standard (public) and supplementary (private/corporate) membership tiers, the center ensures accessibility for a broad range of survivors, from low-income earners to high-level civil servants. According to the institution’s 5-year retrospective service report, approximately 92% of patients’ treated are NHIF beneficiaries, while 2% are covered by other private health insurances (such as Jubilee and Strategy). Only 3% of patients are self-paying (cash), with the remaining 3% receiving care through institutional waivers or social support frameworks for the indigent.

As a specialised facility, Besta follows a standard post-treatment protocol for prostate cancer survivors, which includes routine oncological surveillance consisting of Prostate-Specific Antigen (PSA) monitoring and Physical Examination (Digital Rectal Examination). This specialist-led follow-up is primarily focused on the detection of recurrence and the management of late-onset radiation toxicities, such as complex urological or bowel complications. This standard of care remains consistent with oncology practices across other tertiary or specialised centers in Tanzania [19].

### Study population and sampling

The study population consisted of prostate cancer survivors who had completed their primary definitive treatment phase. While the cohort was heterogeneous regarding prior interventions, including surgery, chemotherapy and androgen deprivation therapy (ADT), the common inclusion criterion was the completion of RT. Because Besta serves as the referral center for RT, this treatment phase served as the standardised marker for ‘completion of active treatment’ within this study. In addition, the study included those survivors who attended routine follow-up appointments between January 2021 and December 2023, with complete medical records, as well as being reachable through telephone. Survivors were excluded if they had documented metastasis, recurrence, secondary malignancies or if they had received RT for palliative purposes only. A census sampling strategy was employed, where all prostate cancer survivors meeting the inclusion criteria during the study period were recruited. While all participants were recruited from Besta Super Specialised Polyclinic during their routine specialist follow-up, the study specifically investigated their concurrent use of PHC facilities. This dual-care approach allowed us to identify survivors who were utilising local district hospitals, health centers or dispensaries for intermediate care between their scheduled specialist appointments at the polyclinic. Records were retrieved from the clinic registry and screened for eligibility.

### Data collection instrument and quality assurance

Data were collected using a structured questionnaire comprising three sections. The first section recorded baseline sociodemographic and clinical data extracted from medical records. The second section assessed survivorship outcomes using the European Organisation for Research and Treatment of Cancer Quality of Life Questionnaire Prostate Cancer Module (EORTC QLQ-PR25), a standardised instrument designed to evaluate symptoms specific to prostate cancer. This tool has been widely validated in multiple languages and settings for assessing urinary, bowel and sexual functioning [20–23]. The third section measured PHC utilisation using a single-item measure consistent with previous survivorship literature [17, 18].

To ensure validity, the EORTC QLQ-PR25 was translated into Swahili and reviewed by the research team to ensure content validity and contextual appropriateness for the Tanzanian setting. The reliability of the PHC utilisation measure was established through a test-retest pilot study involving 29 survivors of pelvic malignancies. This population was selected for the pilot because they share overlapping treatment-related symptoms and survivorship experiences with prostate cancer patients [24]. The measure demonstrated high stability, with a Cohen’s kappa coefficient of 0.97.

### Data collection and variables

Data were collected between May and June 2024 using a combination of medical record extraction and telephone interviews.

Sociodemographic and clinical data: Information on age, education, marital status, occupation, treatment modalities (surgery, chemotherapy, ADT), disease stage and comorbidities was extracted from hospital medical records. Residence was categorised as ‘Rural’ or ‘Urban’ based on the participant’s self-reported district of residence, aligning with the national census administrative designations.

PHC utilisation: During telephone interviews, participants were asked if they had visited a PHC facility (dispensary, health center or district hospital) for follow-up care in the past 12 months. PHC utilisation was defined as the self-reported, any visit to district hospitals or health centers or dispensaries for needs such as the management of treatment-related side effects (such as fatigue or pain), sexual health support and the monitoring of pre-existing comorbidities (e.g., hypertension, diabetes) which may be exacerbated by cancer treatment. Utilisation was measured by whether survivors reported at least one visit to any PHC facility in the past 12 months. For analytic purposes, frequency of visits was categorised into three groups: none (0 visits), occasional (1–2 visits) and frequent (>2 visits). This definition aligns with previous survivorship care literature, where PHC is considered the first point of contact for routine follow up, management of treatment-related side effects and continuity of care in resource-limited settings [14, 15].

Symptom assessment: Survivorship outcomes were assessed using the EORTC QLQ-PR25 module. This standardised tool evaluates symptoms specific to prostate cancer, including urinary, bowel, treatment-related and sexual symptoms. The tool was translated into Swahili and validated for content appropriateness.

### Statistical analysis

Data were analysed using SPSS version 25 (IBM Corp., Armonk, NY, USA). Descriptive statistics, including frequencies, percentages and medians, were used to summarise sociodemographic and clinical characteristics. For regression analysis, continuous and ordinal variables were categorised to enhance interpretability and facilitate the estimation of odds ratios. Age was dichotomised at 60 years. Symptom scores, which are calculated on a 0–100 scale, were binary coded as ‘Normal/Moderate’ versus ‘Severe’ based on the response anchors, where higher scores (corresponding to responses of ‘Quite a bit’ or ‘Very much’) indicated severe symptomatology, consistent with approaches used in previous clinical quality of life studies to define clinically meaningful thresholds [25, 26]. Bivariate analysis was performed using chi-square tests to determine associations between independent variables and PHC utilisation. Variables with a *p*-value < 0.25 in the bivariate analysis were included in the multivariate model.

A hierarchical logistic regression was employed to assess the independent contribution of symptom burden. Block A, containing sociodemographic and clinical variables, was entered first to control for potential confounders. Block B (symptom domains) was subsequently added to determine if symptom burden predicted utilisation over and beyond the variance explained by sociodemographic and clinical factors. Adjusted odds ratios (AOR) with 95% confidence intervals (CI) were calculated, and statistical significance was set at *p* < 0.05.

### Ethical considerations

Ethical clearance was obtained from the Institutional Review Board (IRB) of the Muhimbili University of Health and Allied Sciences (MUHAS). Permission to conduct the study was granted by the administration of Besta Super Specialised Polyclinic. Informed consent was obtained from all participants prior to data collection. To ensure confidentiality, all data were de-identified and stored in password-protected files.

## Results

### Participant selection

**Figure 1.**
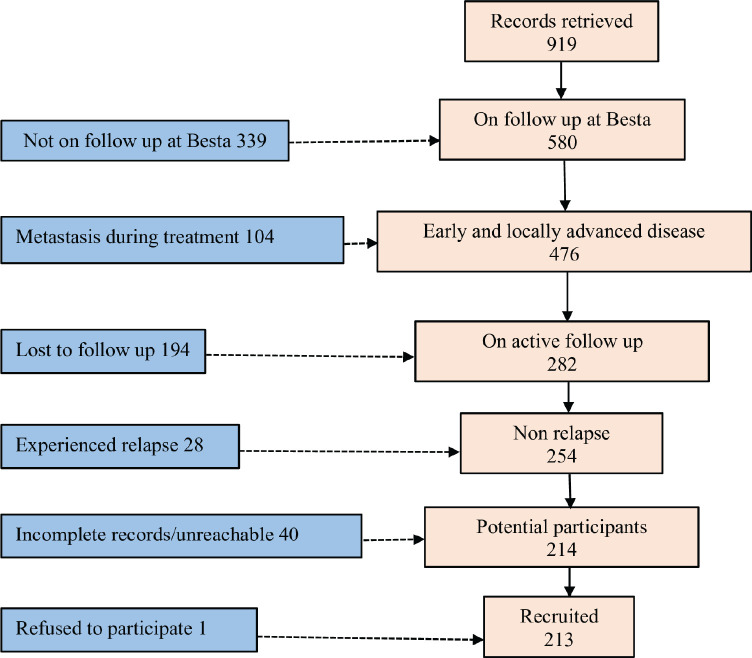
Participant selection flowchart.

A total of 919 RT records were retrieved and screened. Of these, 214 survivors met the inclusion criteria. One survivor declined to participate, resulting in a final sample size of 213 participants, as shown in Figure 1

### Social demographics and clinical profile of the study participants

The median age of participants was 70 years (range: 51–87 years). The majority of participants were aged over 60 years (75.1%), married or cohabiting (88.3%) and resided in rural areas (71.4%). Most participants had a low level of education, with 80.3% reporting primary education or no formal schooling. Subsistence farming (peasants) was the most common occupation (55.4%). The sociodemographic characteristics are detailed in Table 1.

Table 2 describes the clinical characteristics of the study participants. The majority of survivors had been diagnosed with advanced-stage disease (Stage III–IV: 80.3%). Most participants (74.6%) were receiving ADT, while 24.9% had undergone surgery and 39.0% had received chemotherapy. Comorbidities were prevalent, with 38.0% of participants reporting hypertension and 16.0% reporting diabetes. Joint or back pain was a common complaint, reported by 70.9% of survivors.

**Figure 2.**
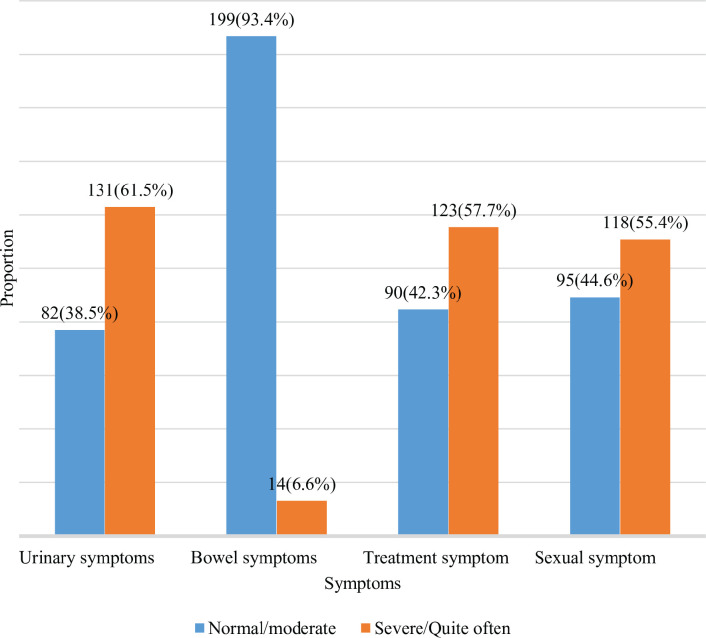
Self-reported symptoms among post-treatment prostate cancer survivors (n = 213).

Across the entire study population (*n* = 213), Figure 2 illustrates the distribution of post-treatment symptoms. While bowel function was largely preserved (93.4% normal/moderate), severe symptoms were prevalent in all other domains.

### PHC utilisation among prostate cancer survivors

**Figure 3.**
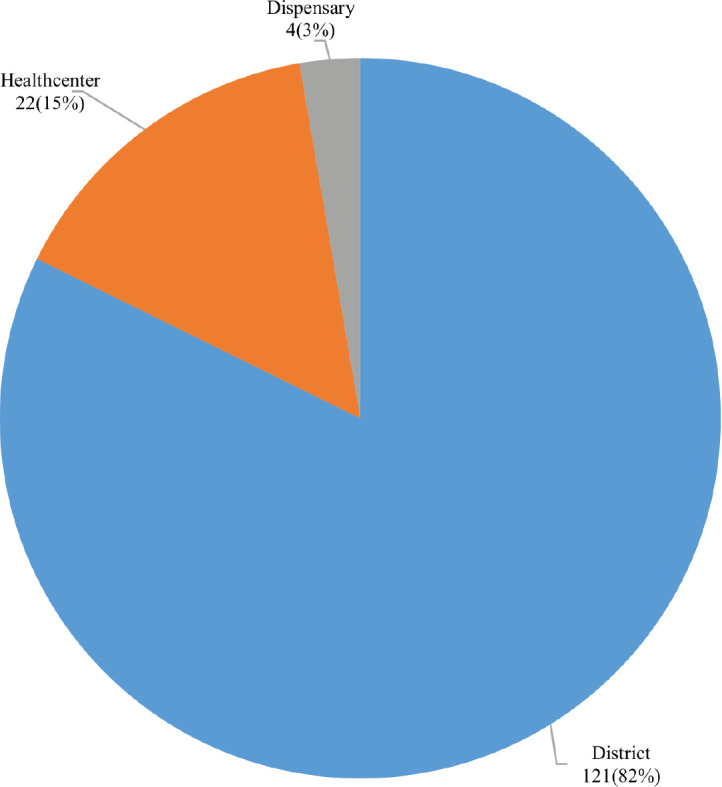
Self-reported PHC utilisation among post treatment prostate cancer survivors (n = 213).

Figure 3 shows the proportion of prostate cancer survivors who reported to have visited PHC at least once in the past 1 year. The overall prevalence of PHC utilisation among survivors was 69.0% (*n* = 147). Among those who utilised PHC services, the majority (82.3%, *n* = 121) visited district hospitals. Regarding the frequency of visits in the past 12 months, 66 participants (31.0%) reported no visits, 25 participants (11.7%) reported occasional visits (1–2 times) and 122 participants (57.3%) reported frequent visits (more than two times).

### Factors associated with PHC utilisation

Table 3 shows the bivariate analysis of the social demographics variables and PHC utilisation. Survivors residing in rural areas had a significantly higher utilisation rate (77.6%) compared to those in urban settings (47.5%; *p* < 0.001). Higher education levels were also associated with increased utilisation (85.7% versus 64.9%; *p* = 0.009).

Table 4 details the bivariate associations between clinical factors, symptom burden and PHC utilisation. Survivors with early-stage disease (I–II) reported significantly higher utilisation (92.9%) compared to those with advanced disease (63.2%; *p* < 0.001). Hypertension and ADT were also significant factors; survivors without hypertension (75.8%; *p* = 0.007) and those not on ADT (81.5%; *p* = 0.022) were more likely to utilise PHC.

Symptom severity played a distinct role in healthcare seeking behaviour. Survivors reporting severe treatment-related symptoms (78.9%; *p* < 0.001) and severe sexual symptoms (79.7%; *p* < 0.001) were significantly more likely to utilise PHC compared to those with moderate symptoms. Conversely, the pattern was reversed for urinary health: survivors with normal or moderate urinary symptoms had higher utilisation rates (80.5%) compared to those with severe urinary complaints (61.8%; *p* = 0.004). Bowel symptoms were not significantly associated with utilisation.

Table 5 presents the hierarchical logistic regression analysis identifying independent predictors of PHC utilisation. In the final model (Block B), rural residence remained a strong predictor, with rural survivors being over 10 times more likely to utilise PHC than urban survivors (AOR = 10.81; 95% CI: 3.98–29.36; *p* < 0.001). Higher education was also associated with increased odds of utilisation (AOR = 5.68; *p* = 0.010).

Severe symptom burden significantly modified the likelihood of PHC use. Survivors reporting severe treatment-related symptoms (AOR = 4.94; *p* < 0.001) and severe sexual symptoms (AOR = 4.97; *p* < 0.001) had significantly higher odds of utilising primary care. Conversely, survivors reporting normal or moderate urinary symptoms were significantly more likely to utilise PHC than those reporting severe urinary complaints (AOR = 3.28; *p* = 0.010). Regarding clinical factors, those with early-stage disease were significantly more likely to use PHC compared to those with advanced disease (AOR = 21.56; *p* < 0.001).

## Discussion

This research set out to measure both the extent and influencing factors of PHC use among post treatment prostate cancer survivors in Tanzania. Findings from this study suggest that 69% of prostate cancer survivors in Tanzania accessed PHC, with most of these users treated in district hospitals. High as it appears, this rate still falls below that of developed nations, where nearly all cancer patients regain access to PHC services in relation to their care [17]. This can be attributed to differences in healthcare structures, as cancer patient survival in Tanzania appears to continue relying on cancer specialists, as opposed to other developed nations that offer comprehensive care [16]. The high utilisation of PHC despite the lack of formal referral mechanisms suggests an informal reliance on local facilities that needs to be structured through shared-care models.

Rural area survivors were found to utilise PHC care significantly more than their counterparts in urban areas, reflecting the profound geographic barriers to accessing centralised specialist care [17, 27]. This reliance highlights a critical disconnect between urban treatment hubs and rural ‘spokes,’ forcing survivors to navigate an informal system for routine support in the absence of institutionalised referral linkages [9, 21]. This finding correlates with studies from other developing nations, where rural populations predominantly utilise local facilities to mitigate the high costs and logistical burdens associated with traveling to distant oncology centers [28, 29]. Such patterns underscore the urgent need for structured shared-care models that formalise the role of rural PHC providers in long-term survivorship management.

A significant association was found between symptom burden and health-seeking behaviour. Patients with severe ADT treatment adverse effects and sexual symptoms demonstrated a higher propensity to seek relative care from PHC. This finding indicates that symptomatic distress, which influences day-to-day functioning, like fatigue, hot flashes and sexual dysfunction, compels survivors to seek immediate care from the facility closest to their residence rather than waiting for scheduled appointments at the tertiary specialists center. This finding conflicts with patterns in developed nations, where complex symptoms usually necessitate a referral to specialists [29]. In the Tanzanian context, survivors navigate a vertical system where, despite being on a centralised follow-up schedule, they rely on PHC for immediate symptomatic relief. However, because this occurs without formal clinical handovers or shared patient data, local practitioners often manage these toxicities in isolation. Hence, it remains unknown if these complex symptoms are managed properly or according to specialised oncological standards.

Conversely, the study observed a unique pattern regarding urinary function, where survivors with normal or moderate urinary symptoms were significantly more likely to utilise PHC than those reporting severe urinary complaints. This finding contrasts with the utilisation patterns observed for sexual and general treatment side effects, where a higher symptom burden drove care-seeking. This disparity may suggest that survivors perceive severe urinary complications, particularly incontinence or obstruction, as complex, specialist-level problems requiring urological intervention at tertiary centers rather than generalist care at the primary level [16]. It is plausible that survivors with severe urinary toxicity bypass lower-level facilities to seek advanced management at the tertiary cancer center, while those with manageable, low-grade urinary symptoms feel comfortable addressing them during routine visits to local health centers [31]. However, in a vertical system lacking formal clinical handovers, this self-triage by patients creates a risk where even manageable symptoms may be overlooked or improperly assessed. This distinction stresses the imperative for standardised referral guidelines to ensure that PHC providers can distinguish between urinary symptoms manageable at the PHC level and those requiring immediate specialist.

Notably, a higher probability of utilising PHC was observed among those with early-stage cancer (Stage I–II) compared to those with advanced disease. This suggests that survivors with localised disease likely perceive their long-term care as manageable within local settings, focusing on routine health maintenance and mild treatment-related toxicities [30, 32]. While these early-stage survivors are clinically stable, they still require vigilant biochemical surveillance through PSA monitoring [32]. This creates a critical opportunity to formalise ‘downward’ referral pathways by empowering PHC providers with standardised survivorship care plans. However, without institutionalised linkages between the specialist ‘hub’ and the PHC ‘spokes,’ these survivors navigate the system informally [21]. Such fragmentation risks a disconnect in oncological surveillance, underscoring the need for integrated digital data sharing to ensure continuity of care.

Educational level was also found to be a determinant in using PHC, with a higher usage among those with a higher educational level. This aligns with existing literature suggesting that higher health literacy among educated survivors empowers them to take greater initiative in navigating the healthcare system and accessing available services [10, 33]. Conversely, the majority of this study population possessed lower educational levels and may face substantial barriers in recognising their clinical needs or understanding the critical role of PHC in their long-term recovery [16]. This disparity underscores the necessity for tailored, low-literacy survivorship education and simplified navigation tools to ensure that less-educated survivors are not disenfranchised from community-level care [5, 7]. Without targeted interventions, the knowledge gap between these cohorts may perpetuate inequities in oncological surveillance and overall health outcomes [33].

The results of this study should be interpreted in light of its limitations. First, the cross-sectional nature of the study prevents establishing causality between independent and outcome variables. Second, using self-reported information for visits to a PHC center can introduce recall bias. Additionally, the inability to reach some survivors via telephone may have introduced selection bias, potentially underrepresenting vulnerable patients. Finally, since this study was facility-based, findings may not be completely generalisable to all patients diagnosed with prostate cancer in Tanzania.

## Conclusion

This study provides the first empirical evidence in Tanzania that PHC facilities already function as a critical, although informal, component of the cancer care continuum. A significant proportion of prostate cancer survivors rely on PHC services for their post-treatment needs, a reliance especially pronounced among individuals living in rural areas and those experiencing severe treatment-related or sexual symptoms. However, the data suggest that survivors with severe urinary complications may bypass these facilities, underlining a potential gap in the capacity of primary care to manage complex urological toxicity. By quantifying the existing informal burden on rural district hospitals, this study provides the empirical foundation for future implementation research. While PHC facilities are already functioning as a critical part of the cancer care continuum, they do so as an informal support system. To optimise the survivorship experience, the healthcare system must transition from fragmented, patient-initiated visits toward structured, integrated shared-care models.

## Conflicts of interest

The authors declare that they have no conflicts of interest.

## Funding

The authors received no financial support for the research, authorship and/or publication of this article.

## Author contributions

RRN served as the principal investigator, conceptualising the study design, conducting data analysis and drafting the manuscript. GPK supervised the entire study and provided critical intellectual guidance. MCS co-supervised the research and assisted in study coordination. YKN (Clinical oversight) contributed to the pre-testing of the data collection tool and led the identification and recruitment of eligible participants from the study site. APT assisted in the data collection process. All authors read and approved the final manuscript.

## Figures and Tables

**Table 1.** Sociodemographic characteristics of prostate cancer survivors (N = 213).

Variables	Frequency (%)
Age	
≤60	53 (24.9)
>60	160 (75.1)
Marital status	
Married	188 (88.3)
Single/widow/divorced	25 (11.7)
Residence	
Rural	152 (71.4)
Township/urban	61 (28.6)
Education level	
Non/primary	171 (80.3)
Secondary/college/Higher	42 (19.7)
Occupation	
Retired/unemployed	95 (44.6)
Peasants	118 (55.4)

**Table 2.** Clinical characteristics of prostate cancer survivors (N = 213).

Variables	Frequency (%)
Stage	
I–II	42 (19.7)
III–IV	171 (80.3)
Surgery	
No	160 (75.1)
Yes	53 (24.9)
ADT	
No	54 (25.4)
Yes	159 (74.6)
Chemo	
No	130 (61.0)
Yes	83 (39.0)
Hypertension	
No	132 (62.0)
Yes	81 (38.0)
Diabetics	
No	179 (84.0)
Yes	34 (16.0)
HIV	
No	182 (85.4)
Yes	31 (14.6)
Back/joint pain	
No	62 (29.1)
Yes	151 (70.9)
ADT, Androgen Deprivation Therapy; HIV, Human Immunodeficiency Virus	

**Table 3.** Association between sociodemographic characteristics and PHC utilisation (n = 213).

Variable	PHC use Yes (Count, %)	PHC use No (Count, %)	p —value
Age			0.000
≤60	25 (47.2%)	28 (52.8%)	
>60	122 (76.3%)	38 (23.8%)	
Marital			0.421
Married	128 (68.1%)	60 (31.9%)	
Single/Widowed/Divorced	19 (76.0%)	6 (24.0%)	
Residence			0.000
Rural	118 (77.6%)	34 (22.4%)	
Township/Urban	29 (47.5%)	32 (52.5%)	
Education level			0.009
Non/Primary	111 (64.9%)	60 (35.1%)	
Secondary/College/Higher	36 (85.7%)	6 (14.3%)	
Occupation			0.024
Retired/Unemployed	58 (61.1%)	37 (38.9%)	
Peasants	89 (75.4%)	29 (24.6%)	

**Table 4.** Clinical factors and symptom burden associated with PHC utilisation (n = 213).

Variable	PHC use Yes (Count, %)	PHC use No (Count, %)	p —value
Stage			0.000
I–II	39 (92.9%)	3 (7.1%)	
III–IV	108 (63.2%)	63 (36.8%)	
Surgery			0.885
No	110 (68.8%)	50 (31.3%)	
Yes	37 (69.8%)	16 (30.2%)	
ADT			0.022
No	44 (81.5%)	10 (18.5%)	
Yes	103 (64.8%)	56 (35.2%)	
Chemotherapy			0.932
No	90 (69.2%)	40 (30.8%)	
Yes	57 (68.7%)	26 (31.3%)	
Hypertension			0.007
No	100 (75.8%)	32 (24.2%)	
Yes	47 (58.0%)	34 (42.0%)	
Diabetes			0.305
No	121 (67.6%)	58 (32.4%)	
Yes	26 (76.5%)	8 (23.5%)	
Back/Joint pain			0.118
No	38 (61.3%)	24 (38.7%)	
Yes	109 (72.2%)	42 (27.8%)	
HIV status			0.314
No	128 (70.3%)	54 (29.7%)	
Yes	19 (61.3%)	12 (38.7%)	
Urinary symptoms			0.004
Normal/moderate	66 (80.5)	16 (19.5)	
Severe/Quite often	81 (61.8)	50 (38.2)	
Bowel symptoms			0.84
Normal/moderate	137 (68.8)	62 (31.2)	
Severe/Quite often	10 (71.4)	4 (28.6)	
Treatment symptoms			<0.001
Normal/moderate	50 (55.6)	40 (44.4)	
Severe/Quite often	97 (78.9)	26 (21.1)	
Sexual symptoms			
Normal/moderate	53 (55.8)	42 (44.2)	
Severe/Quite often	94 (79.7)	24 (20.3)	<0.001
ADT, Androgen Deprivation Therapy; HIV, Human Immunodeficiency Virus			

**Table 5.** Hierarchical logistic regression of factors associated with PHC utilisation (n = 213).

Variables	BLOCK A		BLOCK B	
	95% C.I.for AOR	p value	95% C.I.for AOR	p value
	Lower ─Upper		Lower ─Upper	
Age				
≤60	Ref	1	Ref	1
>60	2.30 (1.00–5.30)	0.051	1.99 (0.75–5.32)	0.170
Residence				
Rural	6.74 (2.97–15.33)	0.000	10.81 (3.98–29.36)	0.000
Township/Urban	Ref	1	Ref	1
Education level				
Non/Primary	Ref	1	Ref	1
Secondary/College/Higher	5.58 (1.80–17.32)	0.003	5.68 (1.52–21.18)	0.010
Occupation				
Retired/Unemployed	Ref	1	Ref	1
Peasants	2.29 (1.10–4.76)	0.027	1.77 (0.78–4.06)	0.175
Stage				
I–II	19.92 (4.26–93.24)	0.000	21.56 (4.23–109.97)	0.000
III–IV	Ref	1	Ref	1
ADT				
No	0.505 (0.17–1.49)	0.216	0.47(0.15–1.49)	0.200
Yes	Ref	1	Ref	1
Hypertension				
No	1.94 (0.94–4.00)	0.072	1.95 (0.85–4.44)	0.113
Yes	Ref	1	Ref	1
Back/Joint pain				
No	1.35 (0.60–3.07)	0.470	1.40 (0.54–3.59)	0.489
Yes	Ref	1	Ref	1
Urinary symptoms				
Normal/moderate			3.28 (1.32–8.15)	0.010
Severe/Quite often			Ref	1
Treatment symptom				
Normal/moderate			Ref	1
Severe/Quite often			4.94 (2.10–11.64)	0.000
Sexual symptom				
Normal/moderate			Ref	1
Severe/Quite often			4.97 (2.11–11.72)	0.000
